# Differential relationships of hepatic and epicardial fat to body composition in HIV

**DOI:** 10.14814/phy2.13386

**Published:** 2017-10-16

**Authors:** Lindsay T. Fourman, Michael T. Lu, Hang Lee, Kathleen V. Fitch, Travis R. Hallett, Jakob Park, Natalia Czerwonka, Julian Weiss, Takara L. Stanley, Janet Lo, Steven K. Grinspoon

**Affiliations:** ^1^ Department of Medicine, Endocrine Division Program in Nutritional Metabolism Massachusetts General Hospital and Harvard Medical School Boston Massachusetts; ^2^ Department of Radiology Cardiac PET MR CT Program Massachusetts General Hospital and Harvard Medical School Boston Massachusetts; ^3^ MGH Biostatistics Center Massachusetts General Hospital Boston Massachusetts

**Keywords:** Ectopic fat, Epicardial fat, Nonalcoholic fatty liver disease (NAFLD), Subcutaneous adipose tissue, Visceral adipose tissue

## Abstract

HIV‐infected patients commonly experience changes in central and peripheral fat content as well as ectopic fat accumulation. However, whether hepatic and epicardial fat stores relate differentially to body composition or how these associations are modified by HIV status has not been well explored. A previously recruited sample of 124 HIV‐infected patients and 58 healthy controls had undergone dual energy X‐ray absorptiometry (DEXA) and computed tomography (CT) from which body composition measures, liver–spleen ratio, and epicardial fat volume were obtained. Unique to the HIV‐infected group, there was a parabolic association between abdominal subcutaneous adipose tissue (SAT) area and liver–spleen ratio (*P *=* *0.03, inflection point 324 cm^2^) such that hepatic fat content was greatest at the extremes of low and high SAT. A quadratic model also closely described the relationship between mean leg fat and liver–spleen ratio among patients with HIV (*P *=* *0.02, inflection point 4.7 kg), again suggesting greater liver fat content with both low and high leg fat. Notably, an analogous relationship of epicardial fat with SAT was not evident among HIV‐infected individuals or healthy controls. In contrast, visceral adipose tissue (VAT) linearly related to both liver–spleen ratio in HIV and epicardial fat volume irrespective of HIV status in multivariable models. In conclusion, our analyses implicate both low and high SAT as risk factors for hepatic fat accumulation in HIV. These findings add to growing evidence of SAT dysfunction in the setting of HIV infection, and highlight key physiologic differences between hepatic and epicardial fat depots.

## Introduction

HIV‐infected individuals may experience complex changes in body composition reflecting the interaction of virus, host, and antiretroviral therapy (ART) superimposed on general population trends (Erlandson and Lake 2016). These alterations include lipohypertrophy of the abdomen, often in the context of a normal body mass index (BMI), and lipoatrophy of the limbs (Martinez 2011). There is no clear association between loss of subcutaneous fat and gain of visceral fat (Bacchetti et al. 2005), suggesting that these processes may occur independently. Although lipoatrophy classically has been ascribed to older ART agents (Martinez 2011), recent clinical trials have shown that 10–20% of treatment‐naïve subjects lose ≥10% of limb fat over 96 weeks on contemporary regimens (McComsey et al. 2016). Mechanisms that may account for HIV lipoatrophy include viral modulation of adipocyte metabolism (Agarwal et al. 2013), and ART‐related mitochondrial toxicity (Martinez 2011).

Aside from changes in fat distribution, generalized gains in visceral and subcutaneous fat in the setting of weight gain have been described among HIV‐infected individuals (McComsey et al. 2016). In one large cohort, 22% of individuals with normal BMI became overweight, and 18% of overweight individuals became obese within 3 years of starting ART (Koethe et al. 2016). Another study reported that trunk and limb fat accumulation continued for years beyond the initial period of treatment initiation, and outpaced the rate of rise among uninfected controls (Grant et al. 2016).

Beyond body composition changes, ectopic fat accumulation is also common among HIV‐infected patients. Nonalcoholic fatty liver disease (NAFLD) comprises a series of histologic abnormalities that stem from hepatocellular triglyceride accumulation including steatosis, steatohepatitis (NASH), fibrosis, and cirrhosis (Qureshi and Abrams 2007). Beyond local liver injury, NAFLD is associated with systemic insulin resistance (Kotronen et al. 2007), dyslipidemia (Kotronen et al. 2007), and coronary artery disease (Park et al. 2016). The estimated prevalence of excess hepatic fat among HIV‐infected individuals is 30–40%, compared to 14–31% among the general population (Guaraldi et al. 2008). Noninvasive imaging evaluation has proven to be a useful surrogate to measure hepatic steatosis (Joy et al. 2003). On noncontrast‐enhanced computed tomography (CT), liver tissue attenuation is reduced by the presence of fat, which translates to lower liver–spleen attenuation ratio with greater hepatic fat fraction (Longo et al. 1993).

Another ectopic fat store, epicardial adipose tissue is uniquely proximal to the heart and its vasculature, and has been postulated to have paracrine effects on the cardiovascular system (Mazurek et al. 2003). Like NAFLD, epicardial fat is associated with coronary artery disease irrespective of HIV status (Guaraldi et al. 2011; Lu et al. 2016). Epicardial fat was increased among HIV‐infected men compared to healthy controls in a preliminary analysis of a subset of our current data (Lo et al. 2010a). Cardiac multidetector row computed tomography (MDCT) parsimoniously captures epicardial fat volume as well as liver and spleen attenuation in the same CT acquisition, thereby allowing for simultaneous assessment of both ectopic fat depots.

While visceral adipose tissue (VAT) has been implicated as a clinical predictor of hepatic and epicardial fat accumulation among HIV‐infected and uninfected groups (Eguchi et al. 2006; Hadigan et al. 2007; Lo et al. 2010a; Guaraldi et al. 2011), relationships between subcutaneous adipose tissue (SAT) such as limb fat and ectopic fat depots have not been well explored. Such associations may be of particular relevance to the care of HIV‐infected patients in whom abnormal loss or gain of SAT is common (Martinez 2011; McComsey et al. 2016). Although NAFLD is not generally regarded as a complication of lipoatrophy in HIV, it is a cardinal feature of genetic lipodystrophy syndromes including Berardinelli–Seip congenital lipodystrophy (Brown et al. 2016). Among HIV‐infected patients, the Fat Redistribution and Metabolic Change in HIV (FRAM) study found that alanine aminotransferase (ALT) varied inversely with trunk SAT (Tien et al. 2008), raising the question of whether low SAT may relate to increased hepatic fat in this population. Beyond relationships between SAT and liver fat, whether physiologic differences between ectopic fat depots manifest as distinct associations with body composition measures or how these relationships are modified by HIV infection has not been well explored.

Here, we investigate the interplay of central and peripheral fat compartments with hepatic and epicardial fat content in a large sample of HIV‐infected patients and healthy controls. We hypothesized that individual ectopic fat depots would relate differentially to body composition parameters. We also postulated that SAT would be associated with ectopic fat uniquely among HIV‐infected patients. Such relationships may provide key clinical and biological insights into the regulation of specific ectopic fat depots and how such regulation is modulated by HIV infection.

## Methods

### Study design

In this cross‐sectional study, we utilized data from a well‐characterized sample of HIV‐infected patients and healthy controls (Lo et al. 2010b; Fitch K et al. 2013; Srinivasa et al. 2017) to explore relationships between body composition and ectopic fat accumulation. Our outcomes of interest were liver–spleen ratio and epicardial fat volume as measured by MDCT. While we have reported relationships of epicardial fat and body composition measures among male (Lo et al. 2010a) and female (Srinivasa et al. 2017) subsets of the current sample, these analyses to characterize novel distinctions between hepatic and epicardial fat stores have not been previously described.

### Participant selection

HIV‐infected patients and healthy controls without known cardiac disease were prospectively recruited from the Boston area between 2006 and 2013 for a study to assess subclinical coronary atherosclerosis with MDCT. Subjects were not enrolled based on body composition or metabolic factors. Patients on ART were required to be on a stable regimen for more than 3 months. Participants who had chronic viral hepatitis or alcohol consumption ≥3 drinks per day were excluded from the current report.

A total of 124 HIV‐infected patients and 58 healthy controls had liver–spleen ratio (HIV^+^ *n* = 102, HIV^−^ *n* = 49) and/or epicardial fat volume (HIV^+^ *n* = 122, HIV^−^ *n* = 58) available for analysis. All subjects gave informed consent to participate. The study was approved by the Institutional Review Boards of Massachusetts General Hospital and Massachusetts Institute of Technology.

### Assessments

All participants underwent a detailed history and physical examination. The weight and height were measured in a fasting state to calculate BMI. Dual energy X‐ray absorptiometry (DEXA, Hologic QDR 4500) was performed to ascertain total body and regional fat mass. The mean leg fat was calculated as an average of fat mass in each leg.

Cardiac MDCT was performed using a Somatom Definition 64‐slice CT scanner (Siemens Healthineers, Forchheim, Germany). As part of the MDCT protocol, a noncontrast‐enhanced CT of the heart was obtained; images of epicardial adipose tissue, the liver, and the spleen were included on this CT acquisition allowing for quantification of these ectopic fat depots. Liver and spleen attenuation were measured by averaging three circular regions of interest with an area of at least 2 cm^2^ within the parenchyma of each organ on different axial slices, taking care to avoid vessels and bile ducts. The ratio of liver to spleen attenuation was then calculated. We previously reported an intraclass correlation coefficient (ICC) of 0.982 using this technique (Lo et al. 2016). Epicardial adipose tissue was defined as tissue within the pericardial sac having attenuation between −195 and −45 Hounsfield units (HU) and quantified in cubic centimeters. We previously reported an ICC of 0.994 as a measure of reproducibility with this technique (Lu et al. 2016). Measures of ectopic fat could not be ascertained in some subjects for technical reasons, such as when the CT scan did not extend far enough caudally to include liver, spleen, and/or lower border of the pericardium. A single slice abdominal CT scan also was acquired at the level of the L4 pedicle to assess SAT and VAT cross‐sectional areas as described elsewhere (Borkan et al. 1982; Lo et al. 2010b).

### Statistical analysis

Demographic and body composition characteristics were compared among HIV‐infected patients and healthy controls with Student's two‐tailed *t*‐test for continuous outcomes and chi‐square test for categorical outcomes. Values are reported as mean ± standard deviation (SD).

Relationships between body composition and ectopic fat accumulation were assessed using a multistep method that began with univariate linear regression. Liver–spleen ratio and epicardial fat volume were treated as continuous outcomes. If there was no linear association between two variables, a quadratic term was added to the model. The inflection point of each significant polynomial expression was calculated algebraically from *y *= c + b*x* + a*x* ^2^ as −b/2a. The polynomial and linear models were compared to assess for fit improvement by the addition of a quadratic term using a likelihood ratio test. We subsequently tested whether fat compartments associated with ectopic fat accumulation in univariate analyses remained significant when controlling for age, gender, BMI, other fat compartments, and duration of ART class (nucleoside reverse transcriptase inhibitor, nonnucleoside reverse transcriptase inhibitor, and protease inhibitor) in multivariable models. These covariates were selected based on their physiologic potential to influence ectopic fat. Among subjects in whom both ectopic fat measurements were available, the linear correlation between liver–spleen ratio and epicardial fat volume was assessed.

Two subjects (one with HIV and one healthy control) were removed prior to analysis after visual inspection of the data and confirmation by formal outlier testing using the Grubbs’ test. A critical value of *P *<* *0.05 was used to designate statistical significance. All statistical analyses were performed using JMP Pro 12.0.1 (SAS Institute Inc., Cary, North Carolina).

## Results

### Liver–spleen ratio

#### Characteristics of participants with liver–spleen ratio available

**Figure 1:**
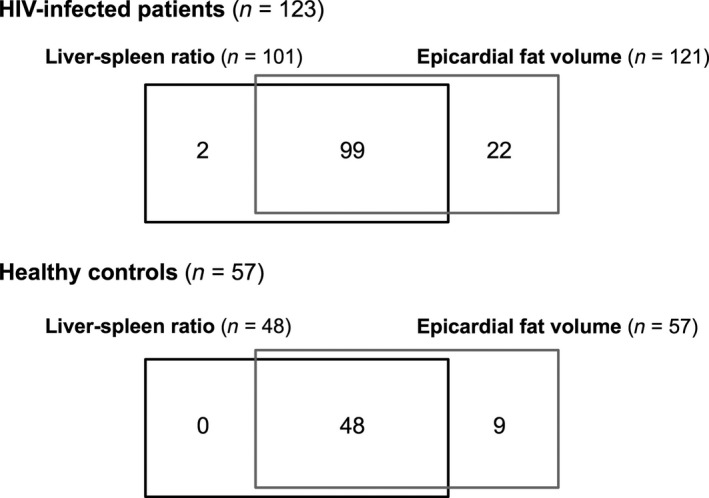
Analysis schema. Overlapping analyses of liver–spleen ratio and epicardial fat are shown for HIV‐infected patients and healthy controls. Values represent the number of subjects with measurements available in each category. All available data were analyzed.

A total of 101 HIV‐infected patients (70% male) and 48 healthy controls (60% male) with liver–spleen ratio were analyzed (Fig. 1). The demographic, immunologic, and metabolic characteristics of the study subjects are described in Table 1. There was no difference in age, gender, race, or alcohol consumption between groups. Individuals with and without HIV had a similar body composition as defined by BMI (27.7 ± 5.3 vs. 27.4 ± 4.9 kg/m^2^, *P *=* *0.73), VAT (154 ± 111 vs. 128 ± 104 cm^2^, *P *=* *0.16), abdominal SAT (234 ± 149 vs. 267 ± 159 cm^2^, *P *=* *0.24), and mean leg fat (3.6 ± 2.2 vs. 4.0 ± 1.9, *P *=* *0.24). Liver–spleen ratio also was comparable between groups (1.40 ± 0.38 vs. 1.42 ± 0.31, *P *=* *0.81).

**Table 1:** Demographic, immunologic, and metabolic characteristics of subjects with and without HIV

	Liver–spleen ratio analysis			Epicardial fat volume analysis		
	HIV^+^ (*n* = 101)	HIV^−^ (*n* = 48)	*P*‐value	HIV^+^ (*n* = 121)	HIV^−^ (*n* = 57)	*P*‐value
Demographics						
Age, years	46.7 ± 8.4	45.9 ± 7.1	0.55	46.7 ± 8.3	45.2 ± 7.3	0.23
Male %	70	60	0.23	66	63	0.70
Race %			0.62			0.37
White	56	58		51	51	
Black	31	29		33	32	
Asian	1	4		1	5	
Hispanic	7	2		10	5	
Native American	4	4		3	4	
Current smoker %	35	40	0.59	35	37	0.81
Current alcohol %	75	69	0.43	70	73	0.66
Current alcohol (drinks/month)	7.6 ± 15	8.8 ± 14	0.63	7.6 ± 17	9.5 ± 15	0.44
Prior IVDU %	7	8	0.75	7	7	0.98
HIV‐related parameters						
Duration of HIV diagnosis (y)	13.9 ± 7.1			14.1 ± 7.1		
CD4 T lymphocytes (cells/mm^3^)	604 ± 328			633 ± 326		
HIV RNA <50 copies/mL (%)	75			79		
Current ART %	83			81		
Duration of ART (y)	6.1 ± 5.1			6.1 ± 5.1		
Body composition parameters						
BMI (kg/m^2^)	27.7 ± 5.3	27.4 ± 4.9	0.73	28.0 ± 5.2	27.3 ± 4.8	0.40
BMI category			0.67			0.48
Overweight %	36	38		40	39	
Obese %	30	23		31	23	
Mean leg fat (kg)	3.6 ± 2.2	4.0 ± 1.9	0.24	3.8 ± 2.1	3.9 ± 1.9	0.58
VAT (cm^2^)	154 ± 111	128 ± 104	0.16	151 ± 108	126 ± 98	0.13
Abdominal SAT (cm^2^)	234 ± 149	267 ± 159	0.24	245 ± 145	259 ± 152	0.55
Liver–spleen ratio	1.40 ± 0.38	1.42 ± 0.31	0.81	1.41 ± 0.39	1.42 ± 0.31	0.85
Epicardial fat volume (cm^3^)	96 ± 57	82 ± 50	0.12	93 ± 55	79 ± 49	0.09

Results are reported as mean ± standard deviation.

IVDU, intravenous drug use; ART, antiretroviral therapy; BMI, body mass index; VAT, visceral adipose tissue; SAT, subcutaneous adipose tissue.

HIV‐infected patients were immunologically well controlled with CD4 count 604 ± 328 cells/mm^3^ and an undetectable viral load in 75%. Self‐reported time from HIV diagnosis was 13.9 ± 7.1 years. The majority of patients (83%) were taking ART at the time of the study for a duration of 6.1 ± 5.1 years.

#### Relationships between body composition and liver–spleen ratio

**Figure 2:**
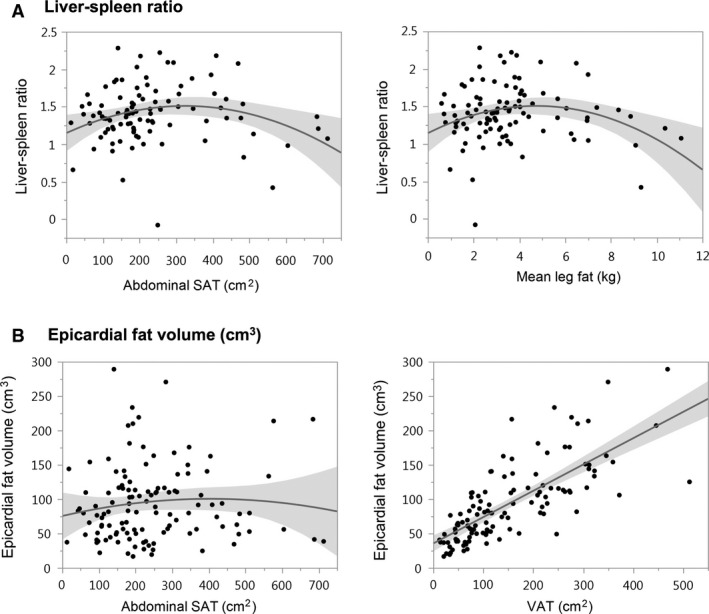
Differential relationships of hepatic and epicardial fat depots to body composition measures among individuals with HIV. (A) Among HIV‐infected individuals, relationships of liver–spleen ratio with abdominal SAT (cm^2^) or mean leg fat (kg) are well described by quadratic models, in which extremes of SAT are associated with a lower liver–spleen ratio (i.e., increased hepatic fat). (B) In contrast, epicardial fat (cm^3^) poorly relates to abdominal SAT (cm^2^) in quadratic (shown) and linear models, whereas there is a strong linear association between epicardial fat (cm^3^) and VAT (cm^2^). Each shaded region represents the 95% confidence band of the regression equation. Liver–spleen ratio = 1.148 + 0.00223(Abd SAT)−3.439e‐6(Abd SAT)^2^, *P *=* *0.03; liver–spleen ratio = 1.142 + 0.153(Mean leg fat)−0.0162(Mean leg fat)^2^, *P *=* *0.02; epicardial fat volume = 75.399 + 0.124(Abd SAT)−0.000153(Abd SAT)^2^, *P *=* *0.55; epicardial fat volume = 35.408 + 0.383(VAT), *P *<* *0.0001. SAT, subcutaneous adipose tissue;VAT, visceral adipose tissue; Abd SAT, abdominal subcutaneous adipose tissue.

Linear and polynomial regression models that explore associations between body composition and liver–spleen ratio are depicted in Table 2A and Figure 2A. Among patients with HIV, there was no linear association between abdominal SAT and liver–spleen ratio (*P *=* *0.89). Instead, the relationship was well described by a quadratic model, whereby liver–spleen ratio was lowest (i.e., liver fat content was highest) at either SAT extreme with an inflection point at 324 cm^2^ (overall *P *=* *0.03, SAT^2^ term *P *=* *0.009). The addition of a quadratic term to the linear regression model significantly improved model fit (*P *=* *0.008).

**Table 2:** Univariate models of body composition parameters and ectopic fat content by depot in HIV‐infected patients and healthy controls

Parameter	Linear model	Quadratic model	Quadratic inflection point	Fit improvement with quadratic model (*P*‐value)
A) Liver–spleen ratio				
HIV‐infected patients				
BMI (kg/m^2^)	*y *=* *1.630−0.00818*x* *P *=* *0.25	*y *=* *−0.247 + 0.122*x*−0.00217*x* ^2^ ***P *** **=** *** *** **0.02**	28 kg/m^2^	**0.01**
Mean leg fat (kg)	*y *=* *1.441−0.00927*x* *P *=* *0.60	*y *=* *1.142 + 0.153*x*−0.0162*x* ^2^ ***P *** **=** *** *** **0.02**	4.7 kg	**0.005**
VAT (cm^2^)	*y *=* *1.504−0.000632*x* *P *=* *0.07	*y *=* *1.472−0.000166*x*−1.112e‐6*x* ^2^ *P *=* *0.18		0.67
Abd SAT (cm^2^)	*y *=* *1.415−3.631e‐5*x* *P *=* *0.89	*y *=* *1.148 + 0.00223*x*–3.439e‐6*x* ^2^ ***P *** **=** *** *** **0.03**	324 cm^2^	**0.008**
Healthy controls				
BMI (kg/m^2^)	*y *=* *1.793−0.0137*x* *P *=* *0.14	*y *=* *3.207−0.113*x *+ 0.00169*x* ^2^ *P *=* *0.21		0.31
Mean leg fat (kg)	*y *=* *1.570−0.0381*x* *P *=* *0.11	*y *=* *1.886−0.208*x* + 0.0185*x* ^2^ *P *=* *0.05		0.05
VAT (cm^2^)	*y *=* *1.480–0.000488*x* *P *=* *0.27	*y *=* *1.432 + 0.000309*x*–2.012e‐6*x* ^2^ *P *=* *0.47		0.58
Abd SAT (cm^2^)	*y *=* *1.527−0.000412*x* *P *=* *0.15	*y *=* *1.717−0.00199*x* + 2.403e‐6*x* ^2^ *P *=* *0.14		0.15
B) Epicardial fat volume (cm^3^)				
HIV‐infected patients				
BMI (kg/m^2^)	*y *=* *−5.817 + 3.524*x* ***P *** **=** *** *** **0.0002**			
Mean leg fat (kg)	*y *=* *104.404−3.206*x* *P *=* *0.17	*y *=* *104.636−3.330*x* + 0.0124*x* ^2^ *P *=* *0.40		0.99
VAT (cm^2^)	*y *=* *35.408 + 0.383*x* ***P *** **<** *** *** **0.0001**			
Abd SAT (cm^2^)	*y *=* *87.438 + 0.0240*x* *P *=* *0.50	*y *=* *75.399 + 0.124*x*−0.000153*x* ^2^ *P *=* *0.55		0.38
Healthy controls				
BMI (kg/m^2^)	*y *= −66.739 + 5.327*x* ***P *** **<** *** *** **0.0001**			
Mean leg fat (kg)	*y *=* *48.406 + 7.911*x* ***P *** **=** *** *** **0.02**			
VAT (cm^2^)	*y *=* *29.376 + 0.392*x* ***P *** **<** *** *** **0.0001**			
Abd SAT (cm^2^)	*y *=* *50.630 + 0.109*x* ***P *** **=** *** *** **0.01**			

*P*‐value for linear or quadratic regression corresponds to overall model. Bold text indicates statistical significance with *P* < 0.05.

BMI, body mass index; VAT, visceral adipose tissue; Abd SAT, abdominal subcutaneous adipose tissue.

As with abdominal SAT, there were parabolic associations between mean leg fat and liver–spleen ratio (overall *P *=* *0.02, leg fat^2^ term *P *=* *0.006), as well as between BMI and liver–spleen ratio (overall *P *=* *0.02, BMI^2^ term *P *=* *0.01) in patients with HIV (Table 2A and Fig. 2A). Liver–spleen ratio again was lowest (i.e., liver fat content was highest) at the extremes of each variable. The inflection point for mean leg fat was 4.7 kg, whereas for BMI it was 28 kg/m^2^. A quadratic model described the data better than a linear model in both cases (mean leg fat *P *=* *0.005, BMI *P *=* *0.01). In contrast, VAT tended to relate linearly to liver–spleen ratio among HIV‐infected individuals (*P *=* *0.07), and inclusion of a quadratic term did not enhance model fit (*P *=* *0.67). When individuals with the lowest VAT (<50 cm^2^) were excluded from the analysis, the linear relationship between VAT and liver–spleen ratio achieved significance (*P *=* *0.04).

Abdominal SAT, VAT, mean leg fat, and BMI were not associated with liver–spleen ratio by linear or polynomial regression analyses among healthy controls. The addition of a quadratic term did not improve model fit for any pair of variables.

#### Multivariable models of body composition and liver–spleen ratio

Two models of liver–spleen ratio were constructed for HIV‐infected and uninfected individuals based on the results of univariate analyses (Table 3). Model 1 included quadratic and linear terms of abdominal SAT, whereas Model 2 included quadratic and linear terms of mean leg fat. Age, gender, BMI, VAT, and duration of ART class were included as covariates in each model. In HIV‐infected patients, squared functions of abdominal SAT (*P *=* *0.0007) and mean leg fat (*P *=* *0.007) were independently associated with liver–spleen ratio. VAT inversely related to liver–spleen ratio (implying a direct relationship between VAT and liver fat content) in Model 1 (*P *=* *0.009), and this relationship tended toward significance in Model 2 (*P *=* *0.05). There was no association between body composition and liver–spleen ratio among healthy controls, consistent with the results of univariate analyses.

**Table 3:** Multivariable models of body composition parameters and ectopic fat content by depot in HIV‐infected patients and healthy controls

Variable	Liver–spleen ratio				Epicardial fat volume (cm^3^)			
	Model 1		Model 2		Model 1		Model 2	
	HIV^+^	HIV^−^	HIV^+^	HIV^−^	HIV^+^	HIV^−^	HIV^+^	HIV^−^
VAT (cm^2^)	**0.009**	0.69	0.05	0.44	**<0.0001**	**<0.0001**	**<0.0001**	**<0.0001**
Abd SAT (cm^2^)	**0.002**	0.64			0.24	0.76		
[Abd SAT (cm^2^)]^2^	**0.0007**	0.35						
Mean leg fat (kg)			0.05	0.25			0.21	0.29
[Mean leg fat (kg)]^2^			**0.007**	0.18				
BMI (kg/m^2^)	0.92	0.59	0.58	0.92	0.58	0.45	0.59	0.17
Age (y)	0.25	0.64	0.14	0.56	0.27	0.07	0.41	0.09
Gender	0.21	0.11	0.65	0.30	**0.02**	0.56	**0.02**	0.89
NRTI duration (y)	0.50		0.44		**0.04**		**0.03**	
NNRTI duration (y)	0.93		0.94		0.16		0.12	
PI duration (y)	0.75		0.68		0.38		0.40	

*P*‐value for each variable is shown. Bold text indicates statistical significance with *P* < 0.05.

VAT, visceral adipose tissue; Abd SAT, abdominal subcutaneous adipose tissue; BMI, body mass index; NRTI, nucleoside reverse transcriptase inhibitor; NNRTI, nonnucleoside reverse transcriptase inhibitor; PI, protease inhibitor.

### Epicardial fat volume

#### Characteristics of participants with epicardial fat volume available

A total of 121 HIV‐infected patients (66% male) and 57 healthy controls (63% male) with epicardial fat volume were analyzed (Fig. 1). Baseline characteristics were similar to those reported for the liver–spleen ratio analysis (Table 1), which was anticipated given that the majority of patients had data available for both analyses. Epicardial fat volume tended to be greater among patients with HIV although this difference did not reach statistical significance (93 ± 55 vs. 79 ± 49 cm^3^, *P *=* *0.09).

#### Relationships between body composition and epicardial fat volume

Linear and polynomial regression models that examine relationships between body composition and epicardial fat volume are displayed in Table 2B and Figure 2B. VAT (*P *<* *0.0001) and BMI (*P *=* *0.0002) were directly and strongly related to epicardial fat volume among HIV‐infected patients in linear regression analyses. There was no linear or quadratic association between abdominal SAT or mean leg fat and epicardial fat volume in this group.

In HIV‐negative individuals, VAT (*P *<* *0.0001), abdominal SAT (*P *=* *0.01), and mean leg fat (*P *=* *0.02), and BMI (*P *<* *0.0001) were positively associated with epicardial fat volume in linear models.

#### Multivariable models of body composition and epicardial fat volume

Two multivariable models were constructed to relate body composition measures to epicardial fat volume among HIV‐infected and uninfected subjects that included VAT, BMI, age, gender, and duration of ART class as covariates (Table**** 3). In addition, Model 1 contained a linear term for abdominal SAT, whereas Model 2 contained a linear term for mean leg fat. Quadratic terms were not included since there was no relationship between a squared body composition parameter and epicardial fat volume in univariate analyses. In HIV‐infected and uninfected groups, VAT was the only body composition measure to associate independently with epicardial fat volume (*P *<* *0.0001).

#### Relationship between epicardial fat volume and liver–spleen ratio

Liver–spleen ratio and epicardial fat volume were not associated among HIV‐infected individuals (*P *=* *0.94) or healthy controls (*P *=* *0.22).

## Discussion

The current study demonstrates that hepatic and epicardial fat depots uniquely relate to body composition, and that these patterns vary by HIV status. In patients with HIV, we show for the first time a parabolic association between abdominal SAT and liver–spleen ratio, suggesting an increase in hepatic fat content at low and high SAT extremes. In contrast, an analogous relationship is not evident for epicardial fat volume among subjects with or without HIV. We also find that VAT linearly relates to liver–spleen ratio among HIV‐infected individuals and epicardial fat volume irrespective of HIV status, which is consistent with previous reports (Hadigan et al. 2007; Lo et al. 2010a; Guaraldi et al. 2011).

Analogous to abdominal SAT, quadratic equations best describe relationships of mean leg fat and BMI to liver–spleen ratio among HIV‐infected patients. Taken together, these models implicate both low and high SAT, the latter a component of generalized obesity, as risk factors for hepatic fat accumulation in HIV. Specifically, liver fat is expected to increase as mean leg fat increases or decreases from an inflection point of 4.7 kg. No correlation is found between body composition parameters and liver–spleen ratio among healthy controls although the smaller sample size of this group may have limited our power to detect an association.

Our report of a relationship between low SAT and increased liver fat in HIV‐infected patients may implicate lipoatrophy as a risk factor for NAFLD in this group. To our knowledge, a link between lipoatrophy and NAFLD previously has not been demonstrated among individuals with HIV. In this regard, prior studies have shown an association between HIV lipodystrophy and increased hepatic fat without further delineating whether liver fat accumulation relates to gain of VAT and/or loss of SAT (Sutinen et al. 2002; Sevastianova et al. 2008). In contrast, hepatic steatosis has been shown to be a complication of lipoatrophy in genetic lipodystrophy syndromes (Brown et al. 2016) although here again quantitative data demonstrating this relationship are limited.

Rather than representing a lack of lipid in otherwise normal adipose tissue, lipoatrophy is characterized by histopathologic abnormalities including reduced adipocyte size, apoptotic changes, macrophage infiltration, and structurally abnormal mitochondria (Giralt et al. 2011). Potential mechanisms to account for the association between lipoatrophy and NAFLD may include deficiency of healthy subcutaneous fat (Gavrilova et al. 2000), as well as inflammatory or hormonal derangements of lipoatrophic adipose tissue (Sevastianova et al. 2008). Unique to HIV, lipoatrophy and hepatic steatosis also may coincide as direct consequences of the virus itself. Mice exposed to viral protein R (Vpr), an HIV accessory protein, demonstrated increased whole‐body lipolysis, reduced white adipose tissue mass, and hepatic steatosis. These animals also exhibited changes in the expression of genes regulated by peroxisome proliferator‐activated receptor *γ* (PPAR*γ*) and glucocorticoid receptor in adipose tissue and PPAR*α* in the liver (Agarwal et al. 2013). Viral protein R has been detected in blood from ART‐suppressed HIV‐infected patients, and thus, it may act as a hormone to induce unfavorable metabolic effects (Agarwal and Balasubramanyam 2015). In addition, HIV‐infected subjects with combined peripheral lipoatrophy and central adiposity were found to have accelerated lipolysis and intrahepatic reesterification compared to uninfected controls, which may suggest the increased flux of free fatty acids from adipose tissue to the liver in this group (Sekhar et al. 2002).

Aside from overt lipoatrophy, the association of low SAT with increased liver fat in HIV may be explained by SAT dysfunction. Specifically, a reduced capacity of SAT to expand may result in the diversion of triglycerides ectopically. Consistent with this hypothesis, a study of biopsy‐proven NAFLD demonstrated that HIV‐positive patients had lower BMI and percentage body fat than HIV‐negative patients (Mohammed et al. 2007). Moreover, in macaques with simian immunodeficiency virus (SIV) compared to uninfected controls, biopsies of SAT and VAT demonstrated a greater CD8^+^/CD4^+^ T‐cell ratio and increased expression of T‐cell activation markers (Damouche et al. 2015). Given that this inflammatory profile resembles that seen in obesity (Nishimura et al. 2009), individuals with HIV‐related SAT inflammation may be short‐circuited toward complications of excess adiposity at a lower BMI.

At the other extreme of body composition, we report an association between high SAT and liver fat in patients with HIV that persists when controlling for VAT and BMI. Moreover, we demonstrate this relationship for subcutaneous fat in both the abdomen and the legs. This finding counters the dogma that SAT is a nonpathologic energy storage depot (Porter et al. 2009) at least in states of profound excess. Previous reports relating elevated SAT to hepatic steatosis in HIV have yielded mixed results. In a large study of HIV‐infected patients, waist circumference was the only aspect of body composition independently to relate to NAFLD, although waist circumference does not distinguish the contributions of SAT or VAT to this association (Guaraldi et al. 2008). Another analysis of HIV‐infected adults found NAFLD to be associated with higher SAT, VAT, and BMI although only VAT correlated with hepatic steatosis following multivariable regression (Hadigan et al. 2007). It is notable that neither of these reports included leg fat in their assessment of body composition, highlighting the novelty of our analysis in this regard.

An emerging literature supports a role for SAT in metabolic syndrome among the general population. In a large sample of Framingham Heart Study participants, the relationship between abdominal SAT and cardiometabolic risk was assessed after stratifying subjects by VAT into sex‐specific tertiles. SAT was positively associated with risk factor prevalence among those in the lower two‐thirds of VAT distribution, implying that SAT is not universally protective and may be detrimental in certain individuals (Porter et al. 2009). Akin to low SAT states, the interplay between excess SAT and increased liver fat may be mediated by adipose tissue inflammation (Nishimura et al. 2009; Le et al. 2011). Among obese individuals from the general population, crown‐like structures, a histological marker of inflammation, were present on abdominal SAT biopsies from those with greater insulin resistance, VAT, and hepatic fat content (Le et al. 2011). It is not known whether the pathologic response of SAT to lipid overload is exaggerated in HIV, but this could account for our detection of a relationship with liver–spleen ratio exclusively in this group.

Among HIV‐infected patients, the parabolic association of SAT with liver fat is distinct to this ectopic fat depot, which may underscore important physiologic differences between hepatic and epicardial fat. Given the unique role of the liver to coordinate energy availability, a limited storage capacity subcutaneously may lead to the preferential shunting of triglycerides to this organ. Accordingly, fat‐specific insulin receptor knock out (F‐IRKO) mice with severe lipoatrophy had a 56% rise in liver weight following an 8‐h feeding. Fasting conversely reduced liver weight and increased ketone levels, implying that hepatic fat is a dynamic energy reserve that can be mobilized (Softic et al. 2016). In contrast to the differential relationships of each depot with SAT, the linear association of VAT with hepatic and epicardial fat may imply that both ectopic fat stores expand in the presence of a positive energy balance. The direct association between VAT and epicardial fat also may be accounted for by the common embryologic origin of these tissues (Sacks and Fain 2007), which could result in similar responses to ART and other factors.

This analysis seeks to characterize unique associations between body composition and ectopic fat stores in the context of HIV, rather than to provide a comprehensive assessment of the diverse contributors to ectopic fat in HIV‐positive and negative individuals. A major strength of our study is its large sample of HIV‐infected men and women, encompassing a broad spectrum of body composition profiles. Another important feature of this report is our detailed, quantitative measurements of central and peripheral body fat compartments by CT and DEXA. Fat wasting has been shown to be detectable by clinical evaluation only once at least 30% of limb fat is lost (Podzamczer et al. 2009), which highlights the superior sensitivity of objective measures over subjective assessment in identifying lipoatrophy. Our analysis also has the flexibility to explore nonlinear models, which may account for the novelty of our findings.

A limitation of this study is its cross‐sectional design from which we cannot assess causality. Additionally, we use CT‐derived liver–spleen ratio as a measure of hepatic fat content rather than a more sensitive technique such as magnetic resonance spectroscopy (MRS) or liver biopsy (Pickhardt et al. 2012). However, it is reassuring that the correlation between liver–spleen ratio by CT and hepatic fat content by MRS has been shown to be excellent (*r* = −0.83) (Longo et al. 1993). Lastly, our cohort contains a smaller number of controls compared to HIV‐infected individuals; relationships within the uninfected group may have been more robust with a larger sample.

In conclusion, the interplay between body composition and ectopic fat varies by depot and HIV status. We show for the first time that hepatic fat content in HIV increases at the extremes of low and high SAT, whereas no comparable relationship with epicardial fat is found, which instead relates more strongly to VAT. This finding has important implications for the care of HIV‐infected patients with low SAT whose heightened risk of NAFLD previously has not been well recognized. Similarly, it is relevant to the growing proportion of obese patients with HIV in whom the role of SAT as a benign storage depot is called into question. In contrast to the divergent associations of each ectopic depot with SAT, we demonstrate that VAT linearly relates to liver fat in HIV‐infected individuals and epicardial fat irrespective of HIV status, which is consistent with previous reports (Hadigan et al. 2007; Lo et al. 2010a; Guaraldi et al. 2011). Taken together, our findings posit important physiologic differences between ectopic fat depots, and suggest a relationship between SAT and liver fat that may be unique to individuals with HIV.

## Conflict of Interest

L.T.F., M.T.L., H.L., K.V.F., T.H., J.P., N.C., J.W., T.L.S., and J.L. have nothing to disclose. S.K.G. has served as consultant to Bristol‐Myers Squibb, Theratechnologies, Navidea Biopharmaceuticals, Novo Nordisk, Merck, and Gilead Sciences, and has received research funding from Theratechnologies, Kowa Pharmaceuticals, Navidea Biopharmaceuticals, Gilead Sciences, Immunex, and Bristol‐Myers Squibb.
